# Genetic PEGylation

**DOI:** 10.1371/journal.pone.0049235

**Published:** 2012-11-08

**Authors:** Seiichi Tada, Takashi Andou, Takehiro Suzuki, Naoshi Dohmae, Eiry Kobatake, Yoshihiro Ito

**Affiliations:** 1 Nano Medical Engineering Laboratory, RIKEN Advanced Science Institute, Wako, Saitama, Japan; 2 Graduate School of Bioscience and Biotechnology, Tokyo Institute of Technology, Yokohama, Kanagawa, Japan; 3 Biomolecular Characterization Team, Chemical Biology Core Facility, Chemical Biology Department, RIKEN Advanced Science Institute, Wako, Saitama, Japan; Berlin Institute of Technology, Germany

## Abstract

Polyethylene glycol (PEG) was genetically incorporated into a polypeptide. Stop-anticodon-containing tRNAs were acylated with PEG-containing amino acids and were then translated into polypeptides corresponding to DNA sequences containing the stop codons. The molecular weights of the PEG used were 170, 500, 700, 1000, and 2000 Da, and the translation was confirmed by mass spectrometry. The PEG incorporation ratio decreased as the molecular weight of PEG increased, and PEG with a molecular weight of 1000 Da was only slightly incorporated. Although improvement is required to increase the efficiency of the process, this study demonstrates the possibility of genetic PEGylation.

## Introduction

Synthetic polymer–protein hybrids have been developed for use as therapeutic proteins or bioreactor enzymes [1]–[10]. Polyethylene glycol (PEG), which is nontoxic, nonimmunogenic, highly soluble in water, and approved by the U.S. Food and Drug Administration (FDA), is very useful in the preparation of therapeutic proteins [11], [12]. Some proteins trigger immune reactions, and proteases and other compounds inside the body can rapidly degrade them and remove them from the body. Many PEGylated protein drugs have been developed since the pioneering work of Abuchowsky et al. on the PEGylation of proteins [13], [14]. Although protein PEGylation has proven very valuable, many of the first-generation PEGylation products suffered from a severe loss in bioactivity [5]. This reduction in activity was mainly attributed to the chain lengths of the attached polymers and the site on the protein to which they are coupled. To overcome this difficulty, a more specific modification was achieved by exploiting the difference in the pKa value of the amine in the lysine side chain or the replacement of lysine residues with other amino acids [15]–[18]. The thiol groups in cysteine residues, the phenol groups of tyrosines, the amide groups of glutamines, or the incorporated His tag have also been used for this specific modification [19]–[29]. The site-specific polymer attachment has also been achieved with the complete synthetic construction of an erythropoietic protein [30], [31], consisting of an α-amino acid polypeptide chain of 166 residues by using native chemical ligation. Recent development of ligation methods has significantly expanded the possibility of bioorthogonal chemistry [32]–[36].

A genetic-encoding approach was first reported in 1989 as an alternative method for the site-specific incorporation of nonnatural amino acids into peptides or proteins [37], [38], and various amino acids have been incorporated in this way [39]–[43]. The method utilizes the UAG codon (the amber nonsense stop codon), which normally directs the termination of protein synthesis, to encode instead a nonnatural amino acid that is loaded onto the complementary tRNA. Deiters et al. [44] made 20 different versions of human growth factor, each of which had a nonnatural amino acid, p-acetylphenylalanine, inserted at a different site using the misacylated tRNA method. They linked a single PEG molecule to each keto group as a posttranslational modification. Johnson et al. [45] enhanced the efficiency of the ribosomal incorporation of unnatural amino acids at multiple sites by RF1 knockout in a live cell. However, it is difficult to precisely insert more than two PEG chains of different lengths or two different unnatural amino acids into each desirable position in one protein molecule using this posttranslational modification method.

**Figure 1:**
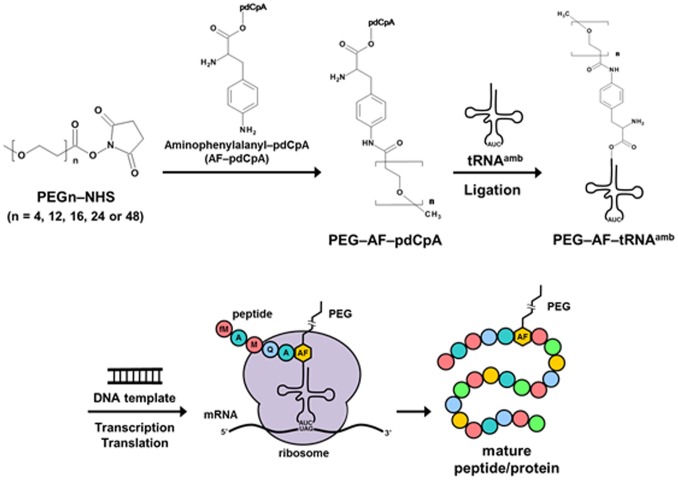
Illustration of the synthesis of polyethylene-glycol-carrying tRNA and the incorporation of PEG into a polypeptide by in vitro translation.

Sisido and co-workers developed a frameshift-suppression method, in which nonnatural amino acids are incorporated into proteins using four-base codon–anticodon pairs instead of the stop codon [46]. Using the four-base codon method, Shozen et al. [47] recently attempted to incorporate a short polyethylene glycol chain into a polypeptide, although its production was not directly confirmed by mass spectrometry, and the incorporation of a long PEG chain was not attempted. Therefore, in this study, we attempted to add tRNAs that recognize a stop codon, to which PEG of various molecular weights was attached, into a translation system, as shown in Figure 1. Here, the incorporation of PEG with a molecular weight of 1000 Da using the in vitro translation system was directly confirmed by mass spectrometry.

## Materials and Methods

### Materials

Ethylene glycol tetramer (PEG4, Mw 170 Da)- or ethylene glycol dodecamer (PEG12, Mw 500 Da)-conjugated aminophenylalanyl tRNAs containing a CUA anticodon were purchased from Protein Express (Chiba, Japan). The single-molecular-weight PEG compounds m-dPEG®_16_–N-hydroxysuccinimide (NHS) ester (PEG16–NHS, Mw 700 Da) and m-dPEG®_24_–NHS ester (PEG24–NHS, Mw 1000 Da) were purchased from Quanta Biodesign (Powell, OH, USA). Methoxypoly(ethylene glycol)–NHS ester of molecular weight 2000 Da, corresponding to PEG48–NHS, was purchased from NOF (Tokyo, Japan). PURESYSTEM classic II for in vitro translation and the PURExpress In Vitro Protein Synthesis Kit were purchased from BioComber (Tokyo, Japan) and New England Biolabs (Ipswich, MA, USA), respectively. Monoclonal anti-FLAG M2 antibody was purchased from Sigma-Aldrich (St Louis, MO, USA).

### Preparation of PEGylated tRNA

The acylated tRNAs were prepared as previously reported [48]. Aqueous solutions of PEG16, 24, and 48 (25 µM, 40 µL) were mixed with aminophenylalanyl–pdCpA (5 µM, 40 µL) in aqueous pyridine-HCl (5 M, pH 5.0, 80 µL). The product is referred to as PEG–AF–pdCpA. After incubation at 37°C for 3 h, PEG–AF–pdCpA was purified by reversed-phase HPLC (Waters XTerra C18; 2.5 µm, 4.6 mm×20 mm) at a flow rate of 1.5 mL/min, with a linear gradient of 0%–100% acetonitrile in 0.1% trifluoroacetic acid (TFA) for 10 min. The PEG–AF–pdCpA products were confirmed by matrix-assisted laser desorption ionization time-of-flight mass spectrometry (MALDI–TOF–MS; Voyager DE-PRO, Applied Biosystems, Foster City, CA, USA): PEG16–AF–pdCpA, calculated 1543.61 for (M–H)^–^, found 1544.09; PEG24–AF–pdCpA, calculated 1895.82 for (M–H)^–^, found 1896.41. PEG–AF–pdCpA was ligated to a yeast phenylalanine tRNA that contained a CUA anticodon and lacked the 3′-terminal dinucleotide. The ligation reaction mixture (20 µL) contained tRNA lacking the 3′ dinucleotide (0.5 nmol), PEG–AF–pdCpA in water (4.4 nmol, 2 µL), HEPES-Na (55 mM, pH 7.5), ATP (1 mM), MgCl_2_ (15 mM), DTT (3.3 mM), BSA (20 µg/mL), and T4 RNA ligase (30 U). After incubation at 4°C for 2 h, potassium acetate (pH 4.5) was added to a final concentration of 0.3 M. PEG4– and PEG12–AF–tRNA were purified with the RNeasy Mini Kit (Qiagen, Hilden, Germany). PEG16–, PEG24–, and PEG48–AF–tRNA were purified by reversed-phase HPLC (Applied Biosystems POROS R2/10; 10 µm, 4.6 mm×100 mm) at a flow rate of 1.0 mL/min, with a linear gradient of 0%–100% acetonitrile in 0.1% triethylammonium acetate for 20 min. The purified PEG–AF–tRNAs were lyophilized and stored at –80°C. For the experiments, the PEG–AF–tRNAs were dissolved in potassium acetate (1 mM, pH 4.5) and stored at –80°C.

### DNA Templates

**Figure 2:**
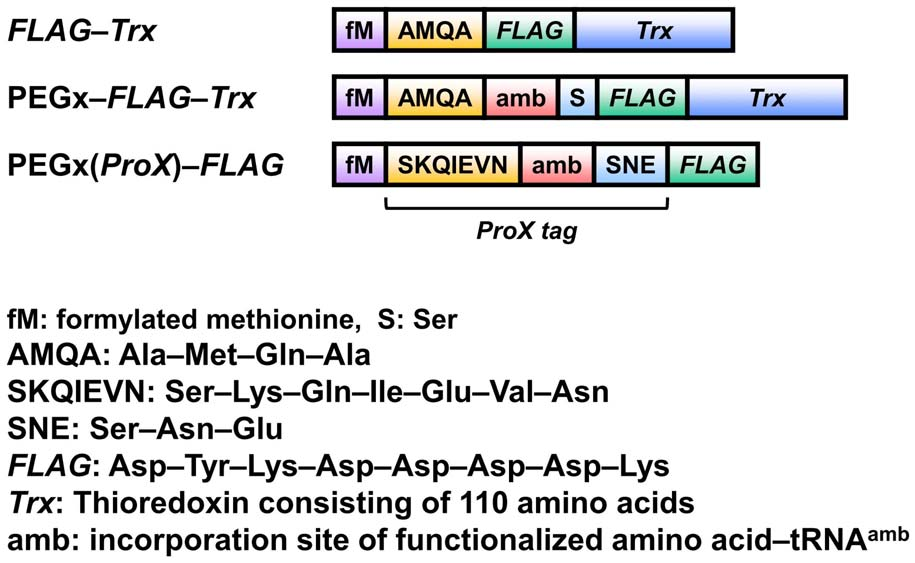
Prepared DNA templates. ProX tag sequence (SKQIEVN–amber-SNE) was contained in PEGx(ProX)–FLAG.

The DNA templates for the cell-free translation of mRNAs that encode the peptide or proteins shown in Figure 2 were constructed by ligating complementary oligonucleotides with the appropriate single-stranded overhangs into pCR2.1 (Invitrogen), previously cut with *Not*I and *Hin*dIII. The plasmid DNA was prepared from ampicillin-resistant transformants and used as the template in PCR reactions with primers corresponding to the T7 RNA polymerase promoter and terminator sequences. The PCR products were purified using the QIAquick PCR Purification Kit (Qiagen) and used as the templates for cell-free translation reactions.

### Cell-free Translation

Cell-free translation was performed using PURESYSTEM classic II (BioComber) or the PURExpress In Vitro Protein Synthesis Kit (New England Biolabs) according to each manufacturer’s protocol. For the incorporation of PEG4, PEG12, PEG16, PEG24, or PEG48, the translation reaction was performed in the presence of 8, 8, 20, 40, or 60 µM, respectively, of each PEG–AF–tRNA. The reaction mixture was incubated at 37°C for 2.5 h, unless otherwise stated.

### Mass Spectra Measurements

The samples were prepared for mass spectrometry as previously reported [39]. The translated peptides were purified from 50 µL reactions using prewashed FLAG M2 agarose (Sigma). After two washes with buffer (50 mM Tris-HCl, 300 mM NaCl, pH 8.0), the peptides were eluted from the matrix with 0.2% TFA. For the mass analysis, the peptides were desalted using ZipTip μ-C18 (Millipore) and mixed with 2,5-dihydroxybenzoic acid or 3-hydroxy-2-pyridinecarboxylic acid as the matrix. The samples were subjected to MALDI–TOF–MS analysis on an Ultraflex spectrometer (Bruker Daltonics) in linear or reflector mode.

### Western Blotting

The reaction mixtures of the translated products (6 µL) were mixed with 3× loading buffer and boiled at 95°C for 3 min, before they were subjected to SDS–PAGE (16.5% acrylamide separation gel and 4% acrylamide stacking gel). The products were detected after western blotting by analysis with horseradish-peroxidase-conjugated anti-FLAG M2 monoclonal antibody (Sigma).

## Results and Discussion

### Preparation of PEGylated Phenylalanyl–tRNAs

**Figure 3:**
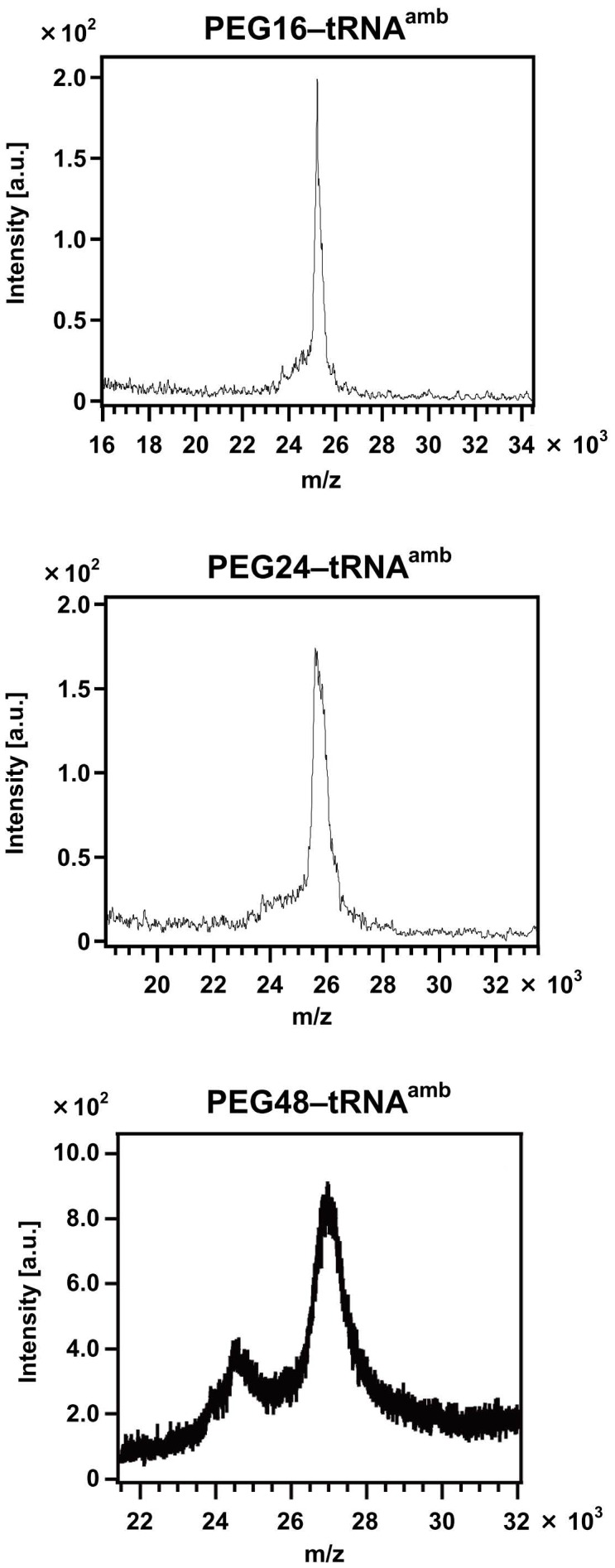
Mass spectra of polyethylene-glycol-carrying tRNAs. The molecular weights of PEG16–tRNA, PEG24–tRNA, and PEG24–tRNA were calculated to be 24485.69, 24838.10, and 25983.45, respectively.

To prepare the nonnatural amino acids carrying PEG chains, PEG was conjugated to aminophenylalanyl–pdCpA. The nonnatural amino acid p-aminophenylalanine has been found to be a good substrate for the in vitro translation system [48]–[51]. The PEG–aminophenylalanyl–pdCpA conjugate was connected to the truncated tRNA with ligase. The synthesis was confirmed by mass analysis, as shown in Figure 3.

### PEGylation Conditions

**Figure 4:**
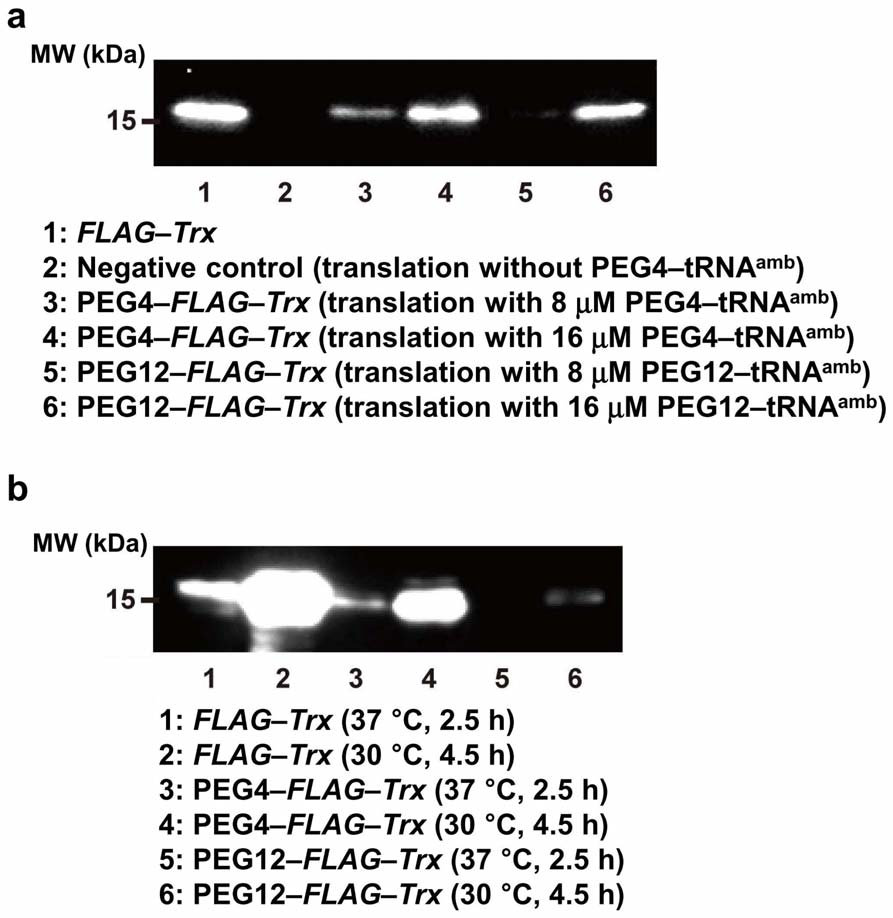
Western blot analysis using anti-FLAG tag antibody of the in vitro translation of thioredoxin (Trx) with an incorporated PEG chain. a. Dependence of protein synthesis on PEG–AF–tRNA concentration. b. PEG-incorporated protein synthesis under different conditions.

Thioredoxin (Trx) was synthesized by in vitro translation using PEG–AF–tRNA (Figure 4). The synthesis of Trx using PEG4 and PEG12 was confirmed by western blotting. The amount of PEGylated Trx produced increased with the addition of increasing amounts of PEG–AF–tRNA (Figure 4a). In the presence of 8 µM PEG–AF–tRNA, more PEGylated product was produced at 30°C for 4.5 h than that at 37°C for 2.5 h (Figure 4b). The yield of Trx protein that contained no amber codons was also increased in the translation reaction at 30°C for 4.5 h. Thus, the efficiency of the cell-free translation was greater at the lower temperature, so the yield of PEGylated protein increased. Bundy and Swartz reported similar results in that the protein yield of a cell-free translation reaction was higher at 30°C for a longer reaction period than at 37°C [52]. They attributed the difference in the protein yields to the rapid reduction of the translation rate at 37°C after 1 h from the beginning of the translation reaction, whereas the reduction in the translation rate at 30°C was relatively slow. The influence of temperature change on cell-free translation and the incorporation of unnatural amino acids requires further investigation.

### PEGylation with Long Chains

**Figure 5:**
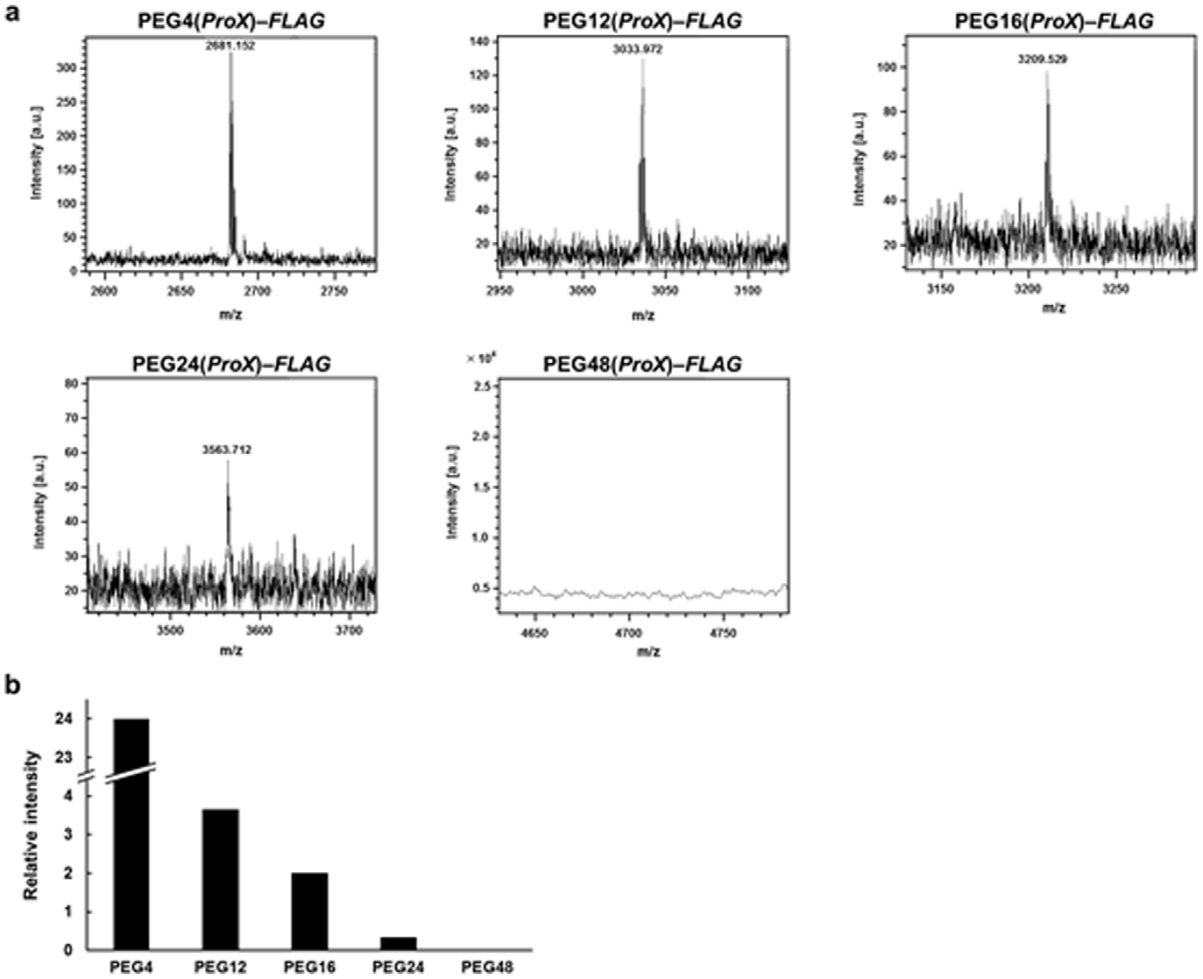
Mass spectrometry analysis of poly(ethylene glycol)-incorporated polypeptide. a. Mass spectra of polypeptide incorporated with poly(ethylene glycol) chain. PEG4–*FLAG*, calculated 2681.177 for (M+H)^+^, found 2681.152; PEG12–*FLAG*, calculated 3033.386 for (M+H)^+^, found 3033.972; PEG16–*FLAG*, calculated 3209.492 for (M+H)^+^, found 3209.529; PEG24–*FLAG*, calculated 3563.890 for (M+H)^+^, found 3563.712; PEG48–*FLAG*, calculated 4709.244 for (M+H)^+^, not found. b. Molecular weight dependence of coefficient of incorporation of poly(ethylene glycol) into a polypeptide by in vitro translation.

Under the conditions described above, FLAG-labeled peptide was prepared by an in vitro translation reaction using PEG4, PEG12, PEG16, PEG24, and PEG48 (Figure 5). To enhance the efficiency of PEG chain incorporation, a peptide sequence containing the ProX tag was used (Figure 2). The sequence of the ProX tag is optimized for the effective incorporation of a nonnatural amino acid into a protein, with minimum irregular product [49]. The translation reactions were performed at 30°C for 7 h and the products were confirmed by mass analysis (Figure 5a). PEG4 or PEG12 was successfully incorporated in the presence of 8 µM PEG–AF–tRNA. However, PEG chains longer than PEG24 were not incorporated. Therefore, the amount of PEG–AF–tRNA in the translation system was increased. PEG16 or PEG24 was incorporated in the presence of 20 µM or 40 µM PEG–AF–tRNA, respectively. However, no incorporation of PEG with a molecular weight of >2000 Da was observed, even in the presence of 60 µM PEG–AF–tRNA.

The dependence of the translational incorporation of PEG on its molecular weight was roughly estimated by mass analysis. Each product peak was compared with a standard peak at a molecular weight of 4364 Da, attributed to ribosomal protein L36 in the cell-free translation system, and the corresponding value was plotted, as shown in Figure 5b. The higher the molecular weight, the less translation product was produced. We inferred that the longer PEG chains sterically hindered the processing of the polypeptide because the tunnel space in the ribosome is narrow. It is also possible that the PEG groups hinder the binding of EF-Tu to the acylated tRNA.

Site-specific PEGylation will be important for the preparation of defined protein drugs. In the future, some modifications to the ribosome should be investigated that enlarge the entrance and exit spaces or increase the binding of EF-Tu to allow the incorporation of longer PEG chains.
